# Alfaxalone Causes Reduction of Glycinergic IPSCs, but Not Glutamatergic EPSCs, and Activates a Depolarizing Current in Rat Hypoglossal Motor Neurons

**DOI:** 10.3389/fncel.2019.00100

**Published:** 2019-03-22

**Authors:** Cora Lau, Prajwal P. Thakre, Mark C. Bellingham

**Affiliations:** Faculty of Medicine, School of Biomedical Sciences, The University of Queensland, Brisbane, QLD, Australia

**Keywords:** motor neuron, alfaxalone, neurosteroid, glycinergic input, glutamatergic input, action potential, hypoglossal motoneurons

## Abstract

We investigated effects of the neuroactive steroid anesthetic alfaxalone on intrinsic excitability, and on inhibitory and excitatory synaptic transmission to hypoglossal motor neurons (HMNs). Whole cell recordings were made from HMNs in brainstem slices from 7 to 14-day-old Wistar rats. Spontaneous, miniature, and evoked inhibitory post-synaptic currents (IPSCs), and spontaneous and evoked excitatory PSCs (EPSCs) were recorded at –60 mV. Alfaxalone did not alter spontaneous glycinergic IPSC peak amplitude, rise-time or half-width up to 10 μM, but reduced IPSC frequency from 3 μM. Evoked IPSC amplitude was reduced from 30 nM. Evoked IPSC rise-time was prolonged and evoked IPSC decay time was increased only by 10 μM alfaxalone. Alfaxalone also decreased evoked IPSC paired pulse ratio (PPR). Spontaneous glutamatergic EPSC amplitude and frequency were not altered by alfaxalone, and evoked EPSC amplitude and PPR was also unchanged. Alfaxalone did not alter HMN repetitive firing or action potential amplitude. Baseline holding current at −60 mV with a CsCl-based pipette solution was increased in an inward direction; this effect was not seen when tetrodotoxin (TTX) was present. These results suggest that alfaxalone modulates glycine receptors (GlyRs), causing a delayed and prolonged channel opening, as well as causing presynaptic reduction of glycine release, and activates a membrane current, which remains to be identified. Alfaxalone selectively reduces glycinergic inhibitory transmission to rat HMNs via a combination of pre- and post-synaptic mechanisms. The net effect of these responses to alfaxalone is to increase HMN excitability and may therefore underlie neuro-motor excitation during neurosteroid anesthesia.

## KEY POINTS

– Motor excitation is a side-effect of alfaxalone anesthesia in animals.
– Alfaxalone at clinically relevant doses causes reduction of glycinergic inhibition of hypoglossal motor neurons and activates a depolarizing inward current.
– Alfaxalone does not alter glutamatergic excitation or motor neuron firing.
– The quality of alfaxalone anesthesia could be improved by preventing its effect on glycinergic neurotransmission.

## Introduction

The neurosteroid anesthetic alfaxalone causes anesthetic effects by positive allosteric modulation of the γ-aminobutyric acid type A receptor (GABA_A_R; Harrison et al., 1987; Lambert et al., 1996; Ziemba et al., 2018). During alfaxalone anesthesia, exaggerated motor responses occur in many species, including rats, mice, cats, dogs, horses and pigs (File and Simmonds, 1988; Keates, 2003; Ferre et al., 2006; Goodwin et al., 2011; Mathis et al., 2012; Lau et al., 2013; Siriarchavatana et al., 2016). This motor excitation is an unwelcome side effect of alfaxalone anesthesia. The glycine receptor (GlyR) mediates inhibitory synaptic transmission between interneurons and motor neurons (MNs; Wu et al., 1997; Maksay et al., 2002; Biro and Maksay, 2004), and reduction of glycinergic inputs to MNs can produce motor excitation (Lynch, 2004). For example, reduction in the conductance of GlyRs causes hyperekplexia, also known as startle disease or stiff-baby syndrome, which manifests as temporary muscle rigidity in response to unexpected stimuli (Lynch, 2004). Tetanus toxin inhibits pre-synaptic exocytosis of glycine, causing muscle rigidity and paroxysmal muscle contractions (Bergey et al., 1987; Williamson et al., 1992; Shin et al., 2012), while strychnine is a heterocyclic alkaloid that potently and specifically blocks GlyRs, thus inhibiting post-synaptic glycinergic activity and producing a characteristic hyper-reflexia, intense muscle spasms and convulsions (Gundlach, 1990; Parker et al., 2011). These effects of down-regulating glycinergic neurotransmission to MNs by various means led us to hypothesize that the motor excitation caused by alfaxalone could be explained by reduction of GlyR transmission to MNs.

Other steroid compounds have markedly different effects on the GlyR, and different neurosteroids have been shown to cause subunit composition-dependent bidirectional modulation of GlyRs (Laube et al., 2002). The synthetic steroid RU5135 possesses convulsant properties and has been identified as a strychnine-sensitive GlyR antagonist (Paul and Purdy, 1992). By contrast, alfaxalone is reported to have no direct effect on GlyRs in cultured neurons (Simmonds, 1983; Hill-Venning et al., 1996) or to weakly potentiate strychnine-sensitive GlyR activity elicited by glycine at a mean EC_50_ of 27.8 μM, without eliciting a response in the absence of glycine (Weir et al., 2004). Similarly, homomeric α1 GlyRs expressed in *Xenopus* oocytes are weakly potentiated by 6 μM alfaxalone on addition of glycine (Mascia et al., 1996). The neurosteroid pregnenolone 3β-sulfate directly modulates GlyRs in chick embryonic spinal cord (Wu et al., 1997) and bi-directional allosteric modulation of the GlyR has been observed with the neurosteroid minaxolone (Biro and Maksay, 2004). However, the effects of neurosteroids on native GlyRs in mammalian MNs have not been reported. It is therefore important to directly determine the effect of alfaxalone on MNs and identify whether alfaxalone produces reduction or potentiation of GlyR-mediated neurotransmission at clinically relevant doses.

While hyperekplexia, tetanus toxicity and strychinine poisoning most notably cause body and facial muscle twitching, deficits in speech and orolingual control are also present. Hypoglossal MNs (HMNs), which control the intrinsic tongue muscles, receive strong glycinergic inputs, and can readily be studied in brainstem slices. We therefore studied the effects of alfaxalone on glycinergic neurotransmission to HMNs, as well as other factors that control HMN excitability, using whole cell patch clamp recording techniques under voltage and current clamp conditions in *in vitro* brainstem slices.

## Materials and Methods

### Slice Preparation

Whole cell recordings (*n* = 71 HMNs) were performed using *in vitro* brainstem slices from Wistar rats of either sex (7–14 days old, *n* = 71), using previously described in methods (Bellingham, 2013). Briefly, rats were anesthetized using sodium pentobarbitone (100 mg/kg IP, Vetcare). When deep anesthesia was established, the rat was swiftly decapitated. The skull, cerebrum, cerebellum and the neck muscles were removed to expose the brainstem. The brainstem was then placed into an ice-cold bath of artificial cerebrospinal fluid (ACSF) for cutting (see “Solutions” section), which was bubbled with carbogen (95%O_2_, 5%CO_2_) to maintain pH at 7.4. Transverse slices at a thickness of 300 μm were cut with a DSK Microslicer DTK-1000 (TED Pella Inc) or a Leica VT1200 (Leica) and incubated for 35–50 min in the same cutting ACSF at 35°C. The slices were then maintained at room temperature (19–21°C) in recording ACSF (see “Solutions” section), bubbled with carbogen.

### Solutions

The ACSF solution used for cutting and initial incubation of slices contained (in mM) 130 NaCl, 26 NaHCO_3_, 3 KCl, 5 Mg Cl_2_, 1 CaCl_2_, 1.25 NaPO_4_, 10 Glucose. The recording ACSF solution was similar to cutting solution except for low MgCl_2_ (1 mM) and high CaCl_2_ (2 mM) concentrations. The patch pipette internal solution contained (in mM) 120 CsCl, 4 NaCl, 4 MgCl_2_, 0.001 CaCl_2_, 10 Cs *N*-2-hydroxyethyl-piperazine-N′-2ethanesulfonic acid (HEPES), 10 Caesium ethylene glycol-bis (β-aminoethyl ether)-N, N, N, N-tetra-acetic acid (EGTA), pH adjusted to 7.2 with CsOH. Similarly, patch pipette internal solution used for recording action potentials contained (in mM) 135 K methyl sulfate, 8 NaCl, 10 HEPES, and 0.3 EGTA, pH adjusted to 7.2 using KOH. Osmolality of both internal solutions was measured with a vapor osmometer (Vapro, Wescor) and was 290–300 mOsm; osmolarity was adjusted with sucrose if required. 3 adenosine 5′-triphosphate (ATP-Mg) and 0.3 guanosine 5-triphosphate-tris (hydroxymethyl) aminomethane (GTP-Tris) was added to the internal solution before use.

### Drugs

DL-2-amino-5-phosphonopentanoic acid (APV, Sigma, 50 μM) and 1,2,3,4-Tetrahydro-6-nitro-2,3-dioxo-benzo[f]quinoxaline-7-sulfonamide (NBQX) disodium salt hydrate (Sigma, 10 μM) were added into the external bath solutions to block N-methyl-D-aspartate (NMDA) and non-NMDA glutamate receptor activity (both DL-α-amino-3-hydroxy-5-methyl-4-isoxazole propionic acid (AMPA) and kainate receptors). 1(S), 9(R)-(-)-Bicuculline methchloride (Sigma, 5 μM) and strychnine (Sigma, 500 nM) solutions were added to the external bath solution to selectively block GABA_A_ receptor and GlyR activity, respectively (O’Brien and Berger, 1999). Strychnine at 20–50 μM was added to simultaneously block both GABA_A_R and GlyR activity (O’Brien and Berger, 1999). Tetrodotoxin (TTX, 1 μM, Alomone) was added to the bath solution to record miniature inhibitory post-synaptic currents (IPSCs). 3-α-hydroxy-5-α-pregna-11, 20-dione (alfaxalone, a generous gift of Jurox Pty Ltd) was dissolved in hydroxypropyl substituted β-cyclodextrin (HPCD, a gift of Jurox Pty Ltd) to a ratio of 1:8 to make a stock concentration of 10 mM alfaxalone, then diluted to the required bath concentration of 10 nM, 30 nM, 100 nM, 300 nM, 1 μM, 3 μM, and 10 μM. For solutions containing 25 μM alfaxalone, a stock solution of 25 mM in HPCD was used. The stock solution solvent (HPCD) was always diluted by a factor of 1,000 or greater in the external bathing solution, and HPCD had no effect when applied alone at these concentrations (data not shown). Application of drugs via the bathing fluid was always for >10 min; the time taken to completely exchange the recording chamber solution was typically <40 s Alfaxalone was applied for a minimum of 6–8 min before measuring changes in synaptic currents or other parameters. The alfaxalone dose response study was only applied to one HMN per slice.

### Electrophysiological Recordings

Brainstem slices were submerged in a mounted microscope chamber with a volume of ~0.5 mL and were continuously superfused with ACSF at a rate of 1.5–2mL/min. Patch electrodes were pulled from thin-walled borosilicate glass capillary tubes without a filament (Vitrex Medical) on a two-stage electrode puller (PP-83, Narishige); patch electrodes had a final DC resistance of 2–3 MΩ when filled with the internal solution and a tip diameter of 1–2 μm by visual inspection. Recordings were performed at room temperature (19–21°C) with the patch electrode connected to the headstage of an Axopatch 1D patch-clamp amplifier (Axon instruments). HMNs were visually identified by their size, shape, location in the hypoglossal motor nucleus (nXII), and whole cell capacitance (>20 pF). Whole cell recordings were obtained by the “blow and seal” method (Stuart et al., 1993), where positive pressure (10–15 kPa) was maintained in the pipette to allow surrounding neuropil to be cleaned away as the pipette tip is guided onto the surface of the target HMN (Bellingham and Berger, 1996).

### Synaptic Recordings

The program pCLAMP 8 (Axon Instruments) was used to apply voltage or current commands and record whole cell currents and measure responses. Spontaneous, miniature and evoked inhibitory postsynaptic current (IPSC) or excitatory PSC (EPSC) activity were recorded with the HMN voltage clamped at a membrane potential of –60 mV. For evoked IPSC or EPSC recordings, a bipolar stimulation electrode [Frederick Haer Company, parallel bipolar electrode, 1 mm spacing and 75 mm length (Cat# PBSA10075)] was placed in the reticular formation ventrolateral to the border of the hypoglossal motor nucleus, and a stimulus current of 0.5–1.1mA and 0.1 ms duration was applied to reliably evoke a pair (150 ms inter-stimulus interval) of IPSCs or EPSCs with consistent first response amplitudes. The recorded signal was amplified (2–50 ×) and low pass filtered with a cut-off frequency of 2 kHz by the Axopatch 1D amplifier before digitization at 10 kHz sampling rate with a 16-bit digitizer (Digidata 1320A, Axon Instruments) and recording on a PC hard disk (Dell Optiplex, running Windows XP Professional). Data was acquired as episodic sweeps of 1.04 s duration for evoked IPSCs and EPSCs, or as continuous data blocks of 2 min duration for spontaneous and miniature IPSCs and EPSCs. For evoked IPSCs and EPSCs, the first stimulus pulse was usually preceded by a short (20 ms) voltage step of −10 mV, to monitor input resistance (Rn) and electrode series resistance. Capacitance and series resistance compensation were applied using the amplifier controls. Series resistance was monitored throughout the experiment and cells were discarded from analysis if it showed more than 10% change from control to drug-induced readings.

### Neurotransmission Study

Control IPSC recordings were performed in the presence of APV and NBQX to determine the proportions of GABA_A_ and GlyR receptors contributing to IPSCs recorded from rat HMNs. After control recordings, 5 μM bicuculline was then bath applied to selectively block GABA_A_R, and both spontaneous and evoked IPSC activity was recorded. This was followed by the addition of 500 nM strychnine to selectively block GlyRs and both spontaneous and evoked IPSCs were recorded. Under the latter conditions, no spontaneous or evoked synaptic activity was present. For all other IPSC recordings, NBQX, APV and bicuculline were included in the bath, except where specifically noted. For all EPSC recordings, bicuculline and strychnine were included in the bath.

### Alfaxalone Dose Response Study

Control recordings were performed in the presence of APV, NBQX and bicuculline to isolate glycinergic IPSCs. A dose response study was performed with alfaxalone bath-applied at 10 nM, 30 nM, 100 nM, 300 nM, 1 μM, 3 μM and 10 μM to HMNs. Both spontaneous (*n* = 7) and evoked (*n* = 8) IPSCs were recorded.

A wash out was attempted after final alfaxalone recordings were made, however, this did not return the recordings to control values. It was particularly difficult to washout alfaxalone at higher concentrations. Considering this, for all other experiments that used high concentration (25 μM) no washout was attempted.

### Action Potential Current Clamp Recordings

Action potentials were recorded in current clamp mode, with the membrane potential manually set at approximately −65 mV by constant injection of baseline current. A current step protocol with a short (40 ms) negative constant current step to monitor bridge balance, followed by a long (400 ms) positive current step family, with positive current injection starting in the subthreshold region and increasing until repetitive action potential firing was evoked at maximal rates. Data was filtered at a low pass cut-off of 10 kHz, and sampled at 50 kHz for these recordings.

### Data Analysis

Data measurements were made with Clampfit 10 software (Axon Instruments). Waveform parameters (amplitude, 10%–90% rise-time, half-width, decay time constant) and baseline holding current (*I*_hold_) were measured, as well as the interval time between IPSCs for spontaneous recordings and input resistance (Rn) for evoked recordings. The paired pulse ratio (PPR) was calculated as the peak amplitude of the averaged second evoked synaptic current divided by the peak amplitude of the averaged first evoked synaptic current. The decay phases of individual evoked synaptic currents were fitted with a mono-exponential function (f(t) = Ae^−t/τ^ + C) where A = amplitude, t = time, *τ* = decay time constant. This data was imported into Excel (Microsoft) and further analyzed with custom written Visual Basic for Applications routines as previously described (Bellingham and Berger, 1996). Drug induced changes were determined by finding the maximal change of 10–12 consecutive responses as compared with the same number of responses for a control average immediately before drug application (Bellingham and Berger, 1996). Data are shown as mean ± 95% confidence interval and statistical significance was determined by one-way repeated measures ANOVA with Dunnet’s or Tukey’s *post hoc* tests as indicated, or paired two-tailed *t*-test and accepted at *P* < 0.05 using GraphPad Prism 7 software.

## Results

### Alfaxalone Decreases Spontaneous Inhibitory Synaptic Currents in HMNs

**Figure 1:**
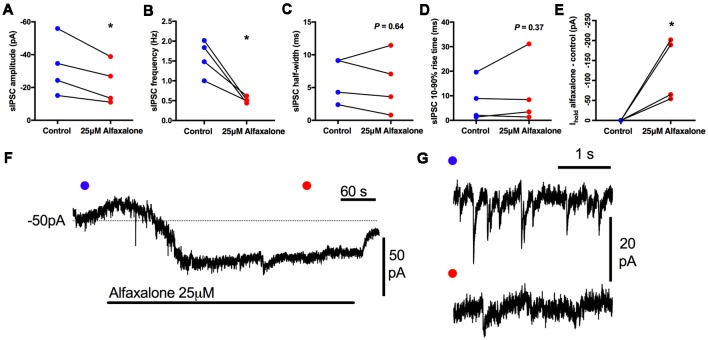
Effects of 25 μM alfaxalone on spontaneous inhibitory post-synaptic currents (IPSCs) and holding current; external solutions contain 1,2,3,4-Tetrahydro-6-nitro-2,3-dioxo-benzo[f]quinoxaline-7-sulfonamide (NBQX) and DL-2-amino-5-phosphonopentanoic acid (DL-APV) to block glutamatergic neurotransmission, but not bicuculline or strychnine. **(A)** Spontaneous IPSC amplitude is significantly decreased by alfaxalone. **(B)** Spontaneous IPSC frequency is significantly decreased by alfaxalone. **(C,D)** Spontaneous IPSC half-width and 10%–90% rise-time are not significantly altered by alfaxalone. **(E)** Holding current at −60 mV is significantly increased in an inward direction by alfaxalone. **(F)** Example of the time course of holding current change with alfaxalone application. Blue and red dots indicate time points of example IPSCs shown in **(G)**. **(G)** Examples of spontaneous IPSC activity before (blue dot, top) and after (red dot, bottom) alfaxalone application. Paired two tail *t*-test, *P < 0.01.

First, we determined whether alfaxalone at a high clinical dose could modulate spontaneous inhibitory transmission to HMNs. Alfaxalone (25 μM) was applied to HMNs in the presence of APV (50 μM) and NBQX (10 μM). Under these recording conditions, IPSCs are composed of both glycine and GABA_A_ neurotransmission. Alfaxalone significantly decreased spontaneous IPSC peak amplitude (Figure 1A) and inter-event interval (Figure 1B), but not 10%–90% rise-time or half-width (Figures 1C,D). IPSC peak amplitude decreased to 69% of control (95% CI 53–75% of control, *P* = 0.0366, Table 1) and markedly decreased IPSC frequency to 33% of control (95% CI 20%–44% of control, *P* = 0.0197, Table 1). The baseline holding current also increased (127% of control, *P* = 0.0479), in an inward direction (Figure 1G; Table 1).

**Table 1:** Effects of alfaxalone (25 μM) on spontaneous inhibitory post-synaptic currents (IPSCs).

Spontaneous IPSC parameter	Control (95% CI)	Alfaxalone (95% CI)	% of control	*P*
Amplitude (pA)	−32.5 (−60.43 to −4.6)	−22.5 (−43.1 to −2.1)	69%	0.0366*
Frequency (Hz)	1.58 (0.87– 2.3)	0.52 (0.4–0.64)	33%	0.0197*
10%–90% Rise-time (ms)	8 (−0.5 to 21.5)	11.1 (−10 to 33)	139%	0.36
Half-width (ms)	6.2 (0.8– 11.7)	5.7 (0.1–13)	92%	0.64
Baseline current (pA)	−68 (−148 to 12)	−196 (−399 to 7)	127%	0.0479*

Mean and 95% CI, paired two-tailed *t*-test, *n* = 4 hypoglossal motor neurons (HMNs). *P < 0.05.

### Inhibitory Neurotransmission to HMNs Is Predominately Glycinergic

**Figure 2:**
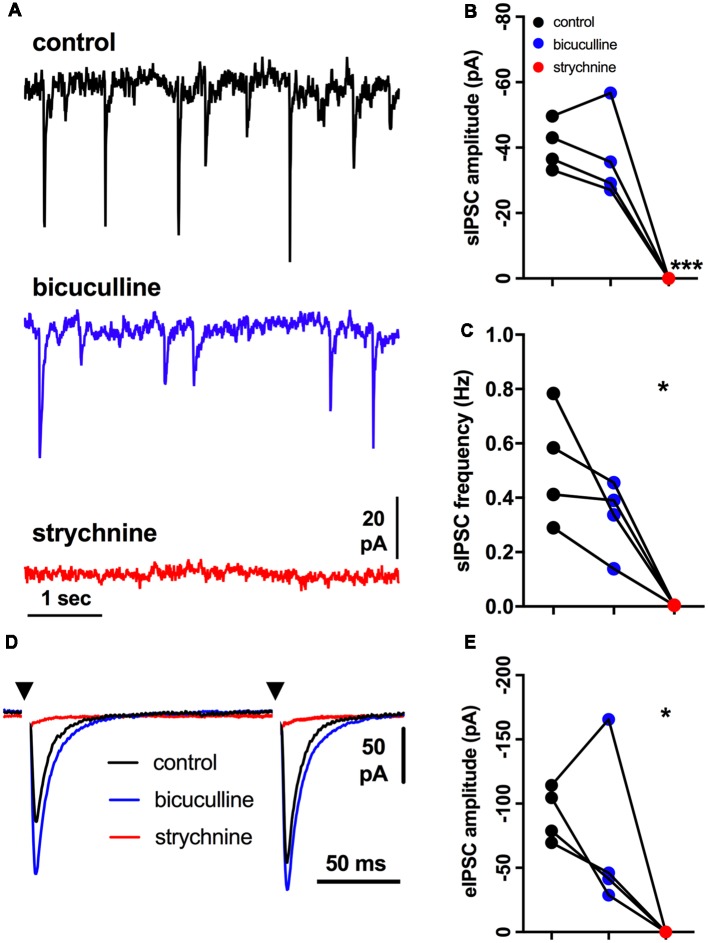
Effects of bicuculline and strychnine on spontaneous IPSCs; external solutions contain NBQX and DL-APV. **(A)** Example traces of spontaneous IPSC activity during control, and after addition of bicuculline (5 μM), then strychnine (500 nM). **(B)** Spontaneous IPSC amplitude is not altered by bicuculline, but is abolished by strychnine addition. **(C)** Spontaneous IPSC frequency is not altered by bicuculline, but is abolished by strychnine addition. **(D)** Example of averaged traces of paired evoked IPSCs showing effects of bicuculline (blue trace), and then strychnine (red trace) addition. **(E)** Evoked IPSC amplitude is not altered by bicuculline, but is abolished by strychnine. Repeated measures one way ANOVA, with Dunnett’s multiple comparisons to control, *P < 0.05, ***P < 0.001.

These results showed that alfaxalone caused suppression of inhibitory neurotransmission to rat HMNs. To determine whether alfaxalone was acting on glycinergic or GABA_A_R mediated synaptic responses, an additional study was performed to determine the contribution of these two receptors to spontaneous and evoked IPSCs in rat HMNs. The GABA_A_R blocker bicuculline, followed by the GlyR blocker strychnine, were bath applied to identify the proportion of IPSCs attributable to the GABA_A_R and the GlyR. Bath application of the GABA_A_R blocker bicuculline (5 μM) did not significantly alter spontaneous or evoked IPSC peak amplitude, rise-time or half-width, or spontaneous IPSC frequency (Figure 2 and Table 2), indicating an insignificant contribution of GABA_A_Rs to IPSCs in rat HMNs under our recording conditions. As the addition of bicuculline did not significantly alter IPSCs, we expected inhibitory synaptic transmission to be glycinergic in nature. The addition of the GlyR blocker strychnine (500 nM) completely blocked evoked IPSCs and all spontaneous IPSCs (Figure 2 and Table 2). This abolition of IPSCs confirmed that the great majority of IPSCs in rat HMNs are attributable to strychnine-sensitive GlyRs. For all further experiments, bicuculline was always applied to definitively exclude any contribution of GABA_A_Rs to IPSCs.

**Table 2:** Effects of bicuculline and strychnine on spontaneous and evoked IPSCs.

Spontaneous IPSC parameter	Control (95% CI)	Bicuculline (5 μM; 95% CI)	*P*	Strychnine (500 nM)	*P*
Amplitude (pA)	−40.6 (−52 to −29)	−37 (−59 to −16)	n.s.	0	***
Frequency (Hz)	0.52 (0.17–0.86)	0.33 (0.11–0.55)	n.s.	0	*
10%–90% Rise-time (ms)	3.7 (1.3–6.2)	3.4 (1.4–5.5)	n.s.	0	n.s.
Half-width (ms)	11.2 (17.6–4.7)	13.0 (0.1–35.8)	n.s	0	n.s.
**Evoked IPSC parameter**					
Amplitude (pA)	−92 (−125 to −58)	−71 (0.1 to −172)	n.s.	0	*
10%–90% Rise-time (ms)	3.4 (0.5–6.3)	3.7 (1.4–6)	n.s.	0	**
Half-width (ms)	8.9 (2.8–14.9)	7.5 (5.1–9.9)	n.s.	0	**

Mean and 95% CI, repeated measures one way ANOVA, with Dunnett’s multiple comparison vs. control, *n* = 4 HMNs. *P < 0.05, **P < 0.01, ***P < 0.0001, n.s. not significant.

### Alfaxalone Causes a Dose-Dependent Decrease in Spontaneous IPSC Frequency, but Does Not Significantly Alter Spontaneous IPSC Size or Shape

**Figure 3:**
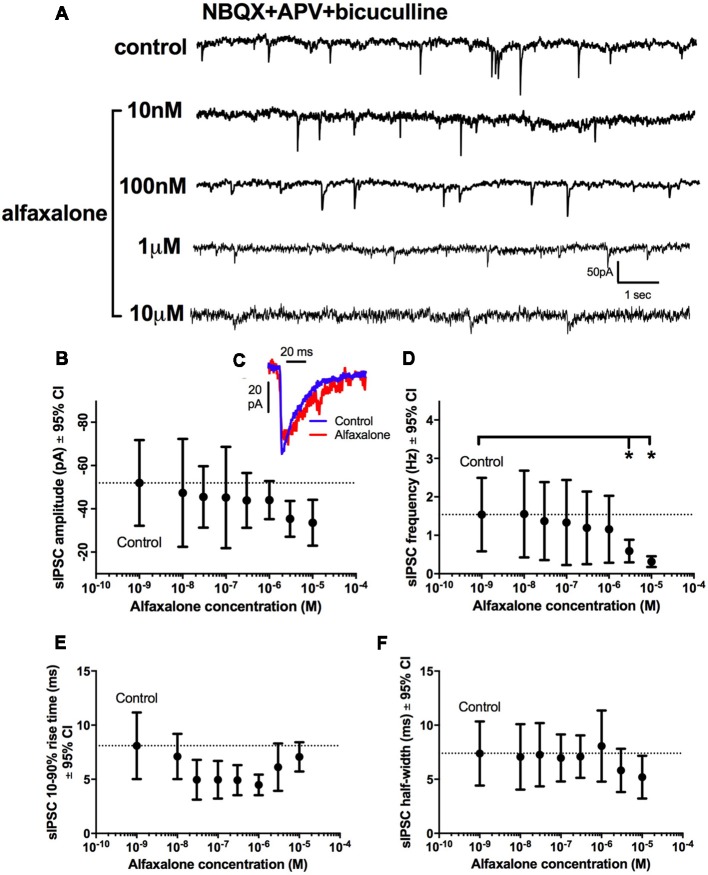
Dose-dependent effects of alfaxalone on spontaneous glycinergic IPSCs; external solutions contain NBQX, DL-APV and bicuculline. **(A)** Example traces of glycinergic IPSCs after addition of 10 nM, 100 nM 1 μM and 10 μM alfaxalone. **(B)** Mean spontaneous glycinergic IPSC amplitude is decreased by increasing alfaxalone concentration. **(C)** Example traces of averaged glycinergic IPSCs before (blue) and after 10 μM alfaxalone (red). **(D)** Mean spontaneous glycinergic IPSC frequency is decreased by increasing alfaxalone concentration. **(E,F)** Spontaneous glycinergic IPSC 10%–90% rise-time and half-width are not altered by increasing alfaxalone concentration. Repeated measures ANOVA, with Dunnett’s post test. *P < 0.05.

A dose response study of the effects of alfaxalone on glycinergic synaptic transmission was carried out to determine whether glycinergic neurotransmission was altered at clinically relevant doses. Pharmacokinetic studies in rat have shown that the minimal effective concentrations of alfaxalone for anesthesia in an adult rat were 1.45 ± 0.79 and 2.06 ± 0.38 mg/L for 2 mg/kg and 5 mg/kg dose ranges respectively (Lau et al., 2013). With a molar mass of alfaxalone at 332.5 g/mol, the minimal effective concentration equivalent in molar concentrations are 4.36 and 6.02 μM, respectively. We thus predicted that suppression of glycinergic neurotransmission would occur at or below these concentrations. A dose dependent decrease in spontaneous IPSC frequency (*P* = 0.003, repeated measures ANOVA with Dunnett’s test) was apparent at 3 μM alfaxalone (*P* < 0.05) and at 10 μM alfaxalone (*P* < 0.05, Figures 3A,D, Table 3). This reduction in glycinergic IPSC frequency suggests that a decrease in presynaptic excitability may reduce glycinergic transmission. Alfaxalone did not significantly alter spontaneous IPSC amplitude (Figures 3B,C), 10%–90% rise-time (Figure 3E) or half-width (Figure 3F) at concentrations up to 10 μM (Table 3).

**Table 3:** Effects of alfaxalone (10 nM–10 μM) on spontaneous glycinergic IPSCs.

Alfaxalone concentration	Amplitude (pA)	Frequency (Hz)	10%–90% rise-time (ms)	Half-width (ms)
Control	−52 (−77 to −28)	1.01 (0.46–2.68)	7.6 (4.2–10.9)	7.2 (3.8–10.7)
10 nM	−47 (−72 to– 22)	1.00 (0.42–2.68)	7.1 (5–9.2)	7.1 (4–10.1)
30 nM	−45.5 (−63 to −28)	0.71 (0.29–2.58)	4.4 (2.9–5.9)	7.1 (3.7–10.5)
100 nM	−45 (−75 to −17)	0.66 (0.12–2.67)	4.4 (3.2–5.5)	6.9 (4.3–9.4)
300 nM	−44 (−58 to −27)	0.64 (0.10–2.3)	4.6 (3.3–5.9)	7 (4.7–9.3)
1 μM	−44 (−52 to −33)	0.57 (0.2–2.22)	4.2 (3.3–5.2)	8 (4.6–11.8)
3 μM	−35 (−44 to −27)	0.44* (0.3–0.88)	6.1 (3.9–8.3)	5.8 (3.8–7.8)
10 μM	−31 (−40 to −21)	0.21* (0.14–0.45)	6.9 (5.4–8.5)	4.8 (2.8–6.9)

Mean ± 95% CI, *n* = 7 HMNs. Repeated measures ANOVA, Dunnett’s test. *P < 0.05.

### Alfaxalone Causes a Reduction in Miniature IPSC Frequency and Amplitude

**Figure 4:**
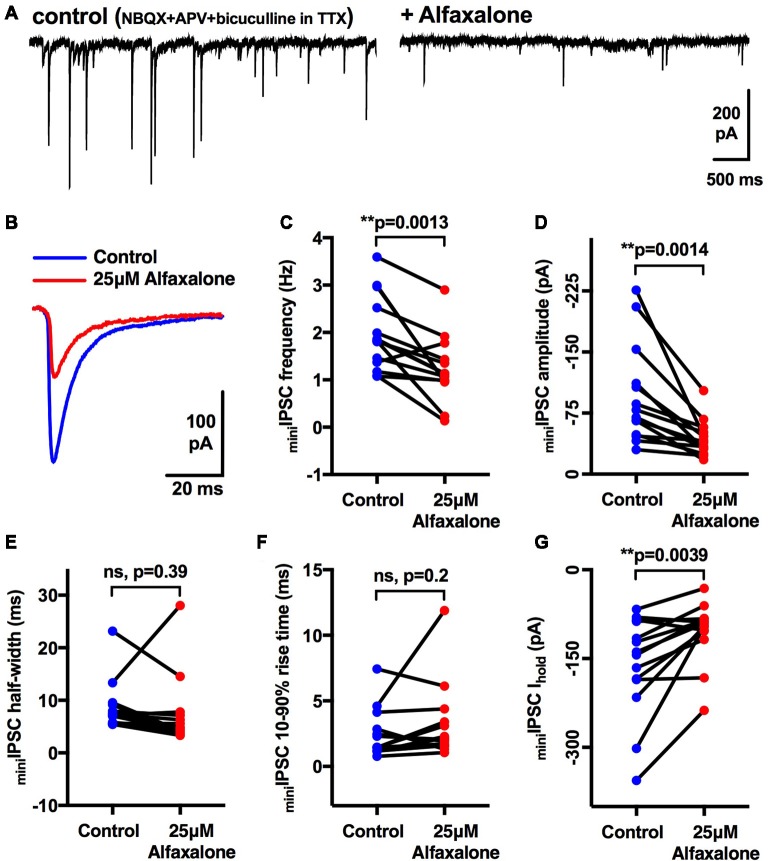
Effects of 25 μM alfaxalone on miniature glycinergic IPSCs; external solutions contain NBQX, DL-APV, bicuculline and tetrodotoxin (TTX). **(A)** Example traces of miniature IPSCs in presence of TTX (1 μM) and after addition of alfaxalone. **(B)** Averaged miniature IPSCs in TTX (blue) and after alfaxalone (red). **(C)** Miniature IPSC frequency is significantly decreased by alfaxalone. **(D)** Miniature IPSC amplitude is significantly decreased by alfaxalone. **(E,F)** Miniature IPSC half-width and 10%–90% rise-time are not altered by alfaxalone. **(G)** Holding current at −60 mV is significantly decreased by alfaxalone. Paired two tail *t*-test, **P < 0.01, n.s. not significant.

As spontaneous IPSC activity is a mixture of action potential-dependent and action potential-independent glycine release, we next determined the effect of a maximal alfaxalone dose (25 μM) on miniature IPSCs, recorded in the presence of TTX (1 μM, Figure 4A). Alfaxalone significantly decreased miniature IPSC frequency to 62% of control (Figure 4C, *P* = 0.0013, *n* = 14 HMNs, Table 4), and reduced miniature IPSC amplitude to 46% of control (Figures 4B,D, *P* = 0.0014, *n* = 14 HMNs, Table 4). Neither rise-time nor half-width of miniature IPSCs were altered by alfaxalone (Figures 4E,F, Table 4), as for spontaneous IPSCs. Interestingly, in this experiment, i.e., in presence of TTX, holding current at −60 mV was significantly shifted in an outward direction, from −161 pA to −104 pA (Figure 4G, *P* = 0.0039, *n* = 14 HMNs).

**Table 4:** Effects of alfaxalone (25 μM) on miniature glycinergic IPSCs.

Alfaxalone concentration	Amplitude (pA)	Frequency (Hz)	10%–90% rise-time (ms)	Half-width (ms)	Iholding (pA)
Control	−95.5 (−130.4 to −60.6)	1.97 (1.5–2.4)	2.4 (1.4–3.5)	8.6 (5.6–11.3)	−160.6 (−209.4 to −111.7)
25 μM	−43.9 (−56.5 to −31.3)	1.21 (0.8–1.6)	3.2 (1.5–4.8)	7.3 (3.5–11.1)	−104.1 (−133.2 to −75)
*P* =	0.0014**	0.0013**	0.2	0.39	**0.0039

Mean and 95% CI, *n* = 14 HMNs. Paired two tailed *t*-test. **P < 0.01.

### Alfaxalone Causes a Dose Dependent Reduction in Evoked IPSC Amplitude and Altered Evoked IPSC Shape

**Figure 5:**
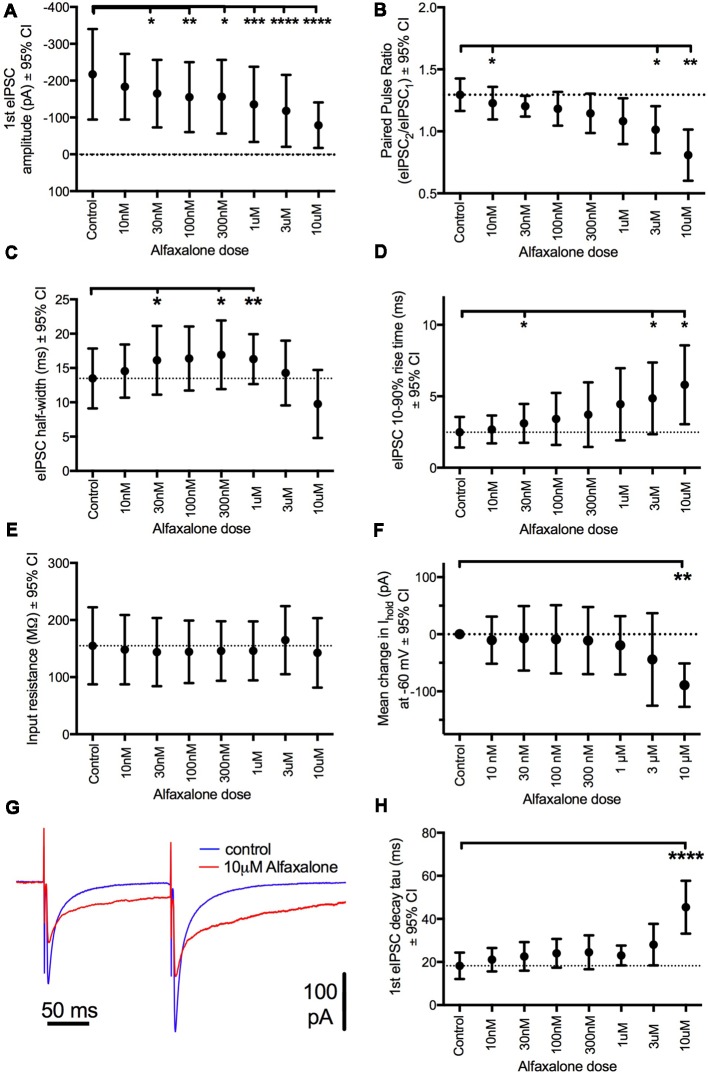
Dose-dependent effects of alfaxalone on glycinergic evoked IPSCs; external solutions contain NBQX, DL-APV and bicuculline. **(A)** Evoked IPSC amplitude is significantly and progressively decreased by increasing alfaxalone concentration. **(B)** The paired pulse ratio (PPR; 2nd IPSC amplitude/1st IPSC amplitude) is significantly and progressively decreased by increasing alfaxalone concentration. **(C)** Evoked IPSC half-width is significantly and progressively altered by increasing alfaxalone concentration. **(D)** Evoked IPSC 10%–90% rise-time is significantly and progressively increased by increasing alfaxalone concentration. **(E)** Input resistance is not altered by alfaxalone. **(F)** Holding current is significantly and progressively increased by increasing alfaxalone concentration. **(G)** Example traces of paired glycinergic IPSCs before (blue) and after (red) alfaxalone. **(H)** The decay time constant of evoked IPSCs is significantly and progressively increased by increasing alfaxalone concentration. Repeated measures ANOVA, with Dunnett’s post test. *P < 0.05, **P < 0.01, ***P < 0.001, ****P < 0.0001.

To further examine alfaxalone effects on glycinergic inputs, we recorded a pair (150 ms inter-stimulus interval) of evoked IPSCs. The evoked IPSC peak amplitude was significantly reduced at 30 nM alfaxalone and evoked IPSC amplitude progressively decreased in a dose-dependent manner, to 36% of control amplitude at 10 μM (Figure 5A and Table 5). This reduction in evoked IPSC amplitude suggests that alfaxalone causes a direct decrease in the sensitivity of postsynaptic GlyRs to glycine. Alfaxalone also slowed the evoked IPSC rise-time in a dose dependent manner. Significantly longer rise-times were observed at 30 nM and 3 μM, with a maximal increase to 234% of control at 10 μM (Figure 5D, Table 5). By contrast, evoked IPSC half-width was not consistently altered by alfaxalone; half-width was significantly increased at 30 nM, 300 nM, and 1 μM, with a maximal increase to 126% of control at 300 nM. Evoked IPSC half-width then decreased, to a minimum of 72% of control at 10 μM (Figure 5C and Table 5). Alfaxalone also produced a significant dose-dependent increase in evoked IPSC decay time constant, which increased to 249% of control at 10 μM (Figure 5H and Table 5, *n* = 8). These results are consistent with postsynaptic modulation of native GlyR channel activity, starting from low nanomolar to micromolar concentrations of alfaxalone.

**Table 5:** Effects of alfaxalone (10 nM–10 μM) on evoked glycinergic IPSCs.

Alfaxalone concentration	1st IPSC amplitude (pA)	1st IPSC 10%–90% rise-time (ms)	1st IPSC half-width (ms)	Paired pulse ratio (2nd/1st)	Input resistance (MΩ)	1st IPSC decay tau (ms)
Control	−217.1 (−340 to −94.2)	2.5 (1.42–3.6)	13.5 (9.1–17.9)	1.3 (1.2–1.4)	155 (87.3–222.4)	18 (12.1–24.5)
10 nM	−183.5 (−272.7 to −94.4)	2.7 (1.7–3.7)	14.6 (10.7–18.4)	1.23 (1.1–1.4)*	148 (87.4–209)	21 (15.7–26.5)
30 nM	−164.8 (−256.5 to −73.1)*	3.1 (1.7–4.5)*	16.1 (11.1–21.1)*	1.2 (1.1–1.3)	144 (84–204)	23 (16–29)
100 nM	−155.2 (−250.2 to −60.1)**	3.4 (1.6–5.2)	16.4 (11.7–21.1)	1.18 (1–1.3)	144 (90–199)	24 (17.4–31)
300 nM	−156.4 (−256.3 to −56.5)*	3.7 (1.5–6)	16.9 (11.9–21.9)*	1.15 (1–1.3)	146 (94–198)	25 (16.7–32.4)
1 μM	−135.6 (−237.6 to −33.6)***	4.4 (2–7)	16.3 (12.7–20)**	1.08 (0.9–1.3)	146 (94.5–198)	23 (18.5–27.7)
3 μM	−118.1 (−215.8 to −20.4)****	4.9 (2.3–7.4)*	14.3 (9.6–19)	1.01 (0.8–1.2)*	165 (105.2–224)	28 (18.5–37.7)
10 μM	−79.0 (−140.9 to −17.2)****	5.8 (3–8.6)**	9.8 (4.8–14.7)	0.81 (0.6–1)**	143 (81.7–204)	45 (33.1–57.7)****

Mean and 95% CI, *n* = 8 HMNs. Repeated measures ANOVA, Dunnett’s multiple comparisons test. *P < 0.05, **P < 0.01, ***P < 0.001, ****P < 0.0001.

### Alfaxalone Decreases Evoked IPSC Paired Pulse Ratio (PPR)

Paired pulse facilitation (PPF) was present in control recordings, and showed a significant dose-dependent decrease throughout the alfaxalone dose range (Figure 5G). IPSC PPF was significantly reduced at 300 nM and 3 μM alfaxalone, and became paired pulse depression at 10 μM alfaxalone (Figure 5B and Table 5). Combined with a decrease in evoked IPSC amplitude, this finding suggests that alfaxalone does not significantly reduce calcium-dependent release of glycine, as a reduction in presynaptic calcium influx should result in increased PPF. The decrease in spontaneous or miniature IPSC frequency was therefore due to a decrease in presynaptic excitability rather than modulation of presynaptic terminal Ca^2+^ influx or basal release probability.

### Alfaxalone Produces an Inward Current Without Altering Input Resistance

During evoked IPSC recording, a dose-dependent inward shift in baseline holding current was observed (*P* = 0.02, repeated measures ANOVA). A significant mean inward shift of −89 pA relative to control was apparent at 10 μM alfaxalone (Figure 5F). However, no significant input resistance changes were observed during alfaxalone application (Figure 5E and Table 5).

### Alfaxalone Does Not Alter Spontaneous Glutamatergic EPSC Amplitude or Frequency

**Figure 6:**
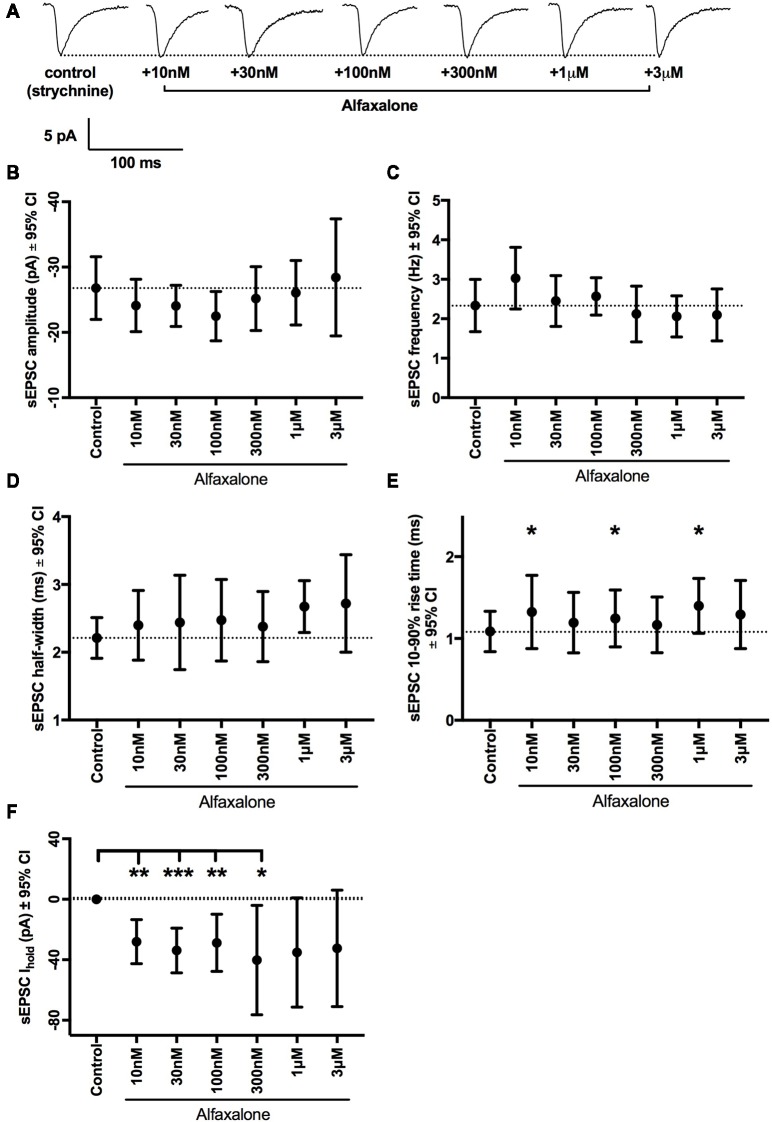
Alfaxalone has no dose-dependent effects on glutamatergic spontaneous excitatory PSCs (EPSCs); external solutions contained 20–50 μM strychnine. **(A)** Example traces of averaged glutamatergic EPSCs after addition of 10 nM, 30 nM. 100 nM, 1 μM, and 3 μM alfaxalone. **(B)** Mean spontaneous glutamatergic EPSC amplitude is not altered by increasing alfaxalone concentration. **(C)** Mean spontaneous glutamatergic EPSC frequency is not altered by increasing alfaxalone concentration. **(D)** Mean spontaneous glutamatergic EPSC half-width is not altered by increasing alfaxalone concentration. **(E)** Mean spontaneous glutamatergic EPSC 10%–90% rise-time is significantly increased by alfaxalone. **(F)** Holding current at −60 mV is significantly increased by 10 nM, 100 nM, and 1 μM alfaxalone. Repeated measures ANOVA, with Dunnett’s post test. *P < 0.05, **P < 0.01, ***P < 0.001.

While reduction of glycinergic neurotransmission to MNs is consistent with neuromotor excitation, the effect of alfaxalone on glutamatergic neurotransmission to MNs has not been investigated. We therefore tested the effects of alfaxalone (10 nM–3 μM) on spontaneous glutamatergic EPSCs (with 20–50 μM strychnine present to block both GABA_A_Rs and GlyRs). Alfaxalone did not significantly alter spontaneous EPSC amplitude or frequency at any concentration (Figures 6A–C, Table 6), but did significantly increase spontaneous EPSC half-width (Figure 6D) at 1 μM (125% of control, *P* = 0.014, *n* = 15 cells) and marginally increased half-width at 3 μM (127% of control, *P* = 0.052, *n* = 9 cells). Spontaneous EPSC rise-time was also significantly increased (Figure 6E) with 10 nM (125% of control, *P* = 0.035, *n* = 13 cells), 100 nM (121% of control, *P* = 0.037, *n* = 13 cells) and 1 μM alfaxalone (138% of control, *P* = 0.031, *n* = 15 cells). The holding current was significantly changed (Figure 6F) in an inward direction at 10 nM (−28 pA, *P* = 0.0013, *n* = 13 cells), 30 nM (−34 pA, *P* = 0.0003, *n* = 13 cells) and 300 nM alfaxalone (−40 pA, *P* = 0.032, *n* = 13 cells).

**Table 6:** Effects of alfaxalone (10 nM–3 μM) on spontaneous glutamatergic excitatory PSCs (EPSCs).

Alfaxalone concentration (*n*=)	Amplitude (pA)	Frequency (Hz)	10%–90% rise-time (ms)	Half-width (ms)
Control (16)	−26.8 (−31.6 to −22)	2.3 (1.7–3)	1.1 (0.8–1.3)	2.2 (1.9–2.5)
10 nM (13)	−24.1 (−28.1 to −20.1)	3.0 (3.8–39.4)	1.3 (0.88–1.8)	2.4 (1.9–2.9)
30 nM (13)	−24.1 (−27.2 to −21)	2.5 (1.8–3)	1.2 (0.8–1.6)	2.4 (1.7–3.1)
100 nM (13)	−22.5 (−26.3 to −18.8)	2.6 (2.1–3)	1.2 (0.9–1.6)	2.5 (1.9–3.1)
300 nM (13)	−25.2 (−30 to −20)	2.1 (1.4–2.8)	1.2 (0.8–1.5)	2.4 (1.9–2.9)
1 μM (15)	−26.1 (−31 to −21)	2.1 (1.5–2.6)	1.4 (1.1–1.7)	2.7 (2.3–3.1)
3 μM (9)	−28.4 (−37.4 to −19.5)	2.1 (1.4–2.8)	1.3 (0.9–1.7)	2.7 (2–3.4)

*Mean ± 95% CI, *n* = 8 HMNs. One way repeated measures ANOVA, Dunnett’s multiple comparisons test*.

### Alfaxalone Does Not Alter Evoked EPSC Amplitude or PPR

**Figure 7:**
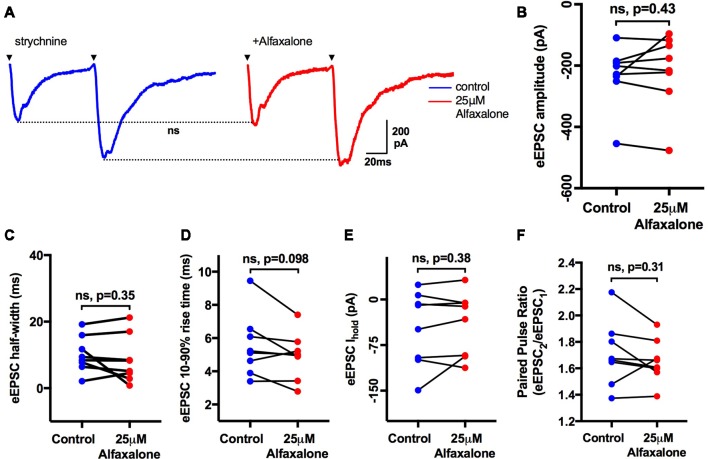
Alfaxalone (25 μM) has no effect on evoked glutamatergic EPSCs or EPSC PPR; external solutions contained 20–50 μM strychnine. **(A)** Example averaged traces of paired glutamatergic evoked EPSCs before and after alfaxalone. **(B)** Evoked EPSC amplitude in not changed by alfaxalone. **(C,D)** Evoked EPSC half-width and 10%–90% rise-time are not altered by alfaxalone. **(E)** Holding current is unchanged by alfaxalone. **(F)** PPR (2nd EPSC amplitude/1st EPSC amplitude) is not changed by alfaxalone. Paired two-tail *t*-test. n.s. not significant.

As alfaxalone elicited subtle effects on spontaneous EPSC shape parameters, while alfaxalone effects on evoked IPSCs were more marked, we also tested the effect of 25 μM alfaxalone on paired (150 ms inter-stimulus interval) evoked EPSCs (Figure 7). Alfaxalone did not significantly alter evoked EPSC amplitude, rise-time or half-width, had no effect on evoked EPSC PPR, and did not significantly alter holding current (Table 7).

**Table 7:** Effects of alfaxalone (25 μM) on evoked glutamatergic EPSCs.

Alfaxalone concentration	1st EPSC amplitude (pA)	1st EPSC 10%–90% rise-time (ms)	1st EPSC half-width (ms)	Paired pulse ratio (2nd/1st)	Baseline holding current (pA)
Control	−232 (−315.3 to −148.4)	5.5 (4–7)	10.1 (5.6–14.7)	1.71 (1.5–1.9)	−47.3 (−98.8 to 4.2)
25 μM	−215 (−317.8 to −113)	4.9 (3.8–6)	8.5 (2.6–14.5)	1.66 (1.52–1.8)	−40.06 (−83.9 to 3.8)
*P* =	0.43	0.1	0.35	0.31	0.38

*Mean and 95% CI, *n* = 8 HMNs. Paired two tailed t-test*.

### Alfaxalone Does Not Alter HMN Repetitive Firing or Action Potential Shape Parameters

**Figure 8:**
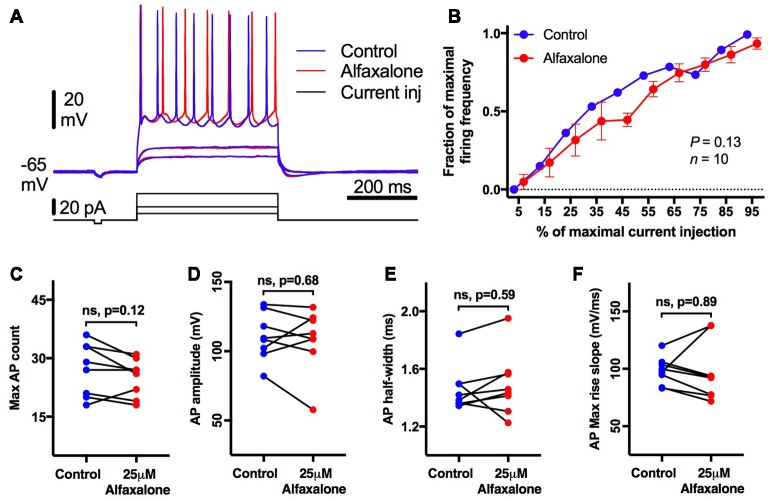
Alfaxalone (25 μM) does not alter hypoglossal motor neuron (HMN) firing or action potentials; external solution contained no blockers. **(A)** Examples traces of subthreshold and repetitive firing responses before (blue) and after alfaxalone (red). **(B)** Firing frequency during increasing current steps is not significantly altered by alfaxalone. **(C)** Maximal action potential count during current injection is unaltered by alfaxalone. **(D,E)** Action potential amplitude and half-width are unaltered by alfaxalone. **(F)** Maximum rise slope of the action potential is not changed by alfaxalone. **(B)** Repeated measures ANOVA. **(C–F)** Paired two-tail *t*-test. n.s. not significant.

As alfaxalone had significant effects on HMN holding current at clinically relevant concentrations, we also examined whether HMN intrinsic excitability and firing was altered by alfaxalone. HMNs were current-clamped at −65 mV, and a series of positive current steps (400 ms duration) were injected to evoke repetitive action potential firing (Figure 8A). After alfaxalone (25 μM), the maximal number of action potentials evoked by current injection was unchanged (Figure 8C), and the firing frequency—current injection curve (Figure 8B) was not significantly different. The amplitude, half-width and maximal rise slope of the first evoked action were also unchanged after alfaxalone (Figures 8D–F, and Supplementary Information).

## Discussion

### Alfaxalone Causes Glycinergic IPSC Inhibition at Clinically Relevant Doses

Neuromotor excitation, manifested as muscle twitching and rigidity, often occurs during alfaxalone anesthesia in animals (File and Simmonds, 1988; Keates, 2003; Ferre et al., 2006; Goodwin et al., 2011; Mathis et al., 2012; Lau et al., 2013; Siriarchavatana et al., 2016). Hyperekplexia, tetanus toxicity or strychnine poisoning, all caused by selective suppression of GlyR-mediated synaptic inhibition of MNs, are similar responses. Here, we show that the neurosteroid alfaxalone produces a selective suppression of glycinergic inhibition of rat HMNs at clinically relevant doses, providing a plausible mechanism for neuromotor excitation during neurosteroid anesthesia. Our results confirmed that alfaxalone caused suppression of glycinergic synaptic transmission to brainstem MNs in a dose-dependent manner. A decrease in evoked IPSC amplitude was observed from 30 nM, increased evoked IPSC rise-time from 1 μM, and decreased IPSC frequency from 3 μM. These changes were apparent below 4–6 μM, the plasma levels required for anesthesia with alfaxalone. In addition, alfaxalone elicited an inward current shift and increased evoked IPSC decay time constant at 10 μM. These data show that modulation of glycinergic transmission occurs at alfaxalone concentrations below those necessary to produce anesthetic immobilization, consistent with motor excitation occurring during recovery from anesthesia with alfaxalone.

Several neurosteroids, including alfaxalone, have been reported to positively or negatively modulate GlyRs (Paul and Purdy, 1992; Mascia et al., 1996; Wu et al., 1997; Laube et al., 2002; Biro and Maksay, 2004; Weir et al., 2004). Here, we show that alfaxalone produces a dose-dependent reduction of native glycinergic IPSCs in rat HMNs, at or below clinically relevant doses, with some effects present at nanomolar concentrations and most effects present at low micromolar concentrations. The minimum plasma concentration of alfaxalone to maintain sleep in rats is 4–6 μM (Lau et al., 2013), similar to that required for human anesthesia (Harrison and Simmonds, 1984). Our results are broadly compatible with postsynaptic modulation of GlyR channel activity, coupled with a decrease in presynaptic release of glycine at clinically relevant alfaxalone concentrations.

### Inhibitory IPSCs in HMNs Are Predominantly Glycinergic

The hypoglossal motor nucleus contains one of the highest GlyR densities within the central nervous system (Singer et al., 1998; Singer and Berger, 1999, 2000), suggesting that glycinergic synaptic transmission contributes to the majority of IPSC activity in HMNs. However, glycinergic synaptic transmission to HMNs undergoes changes during postnatal development (Singer et al., 1998; Singer and Berger, 2000), while co-transmission of GABA and glycine from the same presynaptic vesicle occurs in both brainstem and spinal cord MNs, where dual component miniature IPSCs with GABAergic and glycinergic components were observed (Jonas et al., 1998; O’Brien and Berger, 1999; Mitchell et al., 2007). It has previously been shown that 5 μM bicuculline blocked 97% of GABA_A_R responses and only 6% of GlyR responses, while 500 nM strychnine blocked 97% of GlyR responses and only 11% of GABA_A_R mediated responses in neonatal Sprague-Dawley rat HMNs (O’Brien and Berger, 1999). We found that spontaneous or evoked IPSCs were not significantly altered by 5 μM bicuculline, suggesting minimal GABAergic influence on neonatal rat HMNs. The addition of 500 nM strychnine in the presence of bicuculline abolished evoked IPSCs and spontaneous IPSC activity, confirming that the inhibitory synaptic activity in neonatal rat HMNs was predominantly glycinergic.

### Alfaxalone Decreases Spontaneous IPSC Amplitude and Frequency

Alfaxalone decreased spontaneous IPSC amplitude at 25 μM and decreased spontaneous IPSC frequency at 3–25 μM. These effects are consistent with postsynaptic modulation of GlyR activity and presynaptic reduction in glycine release. We note that we did not specifically test the effects of alfaxalone at 25 μM on spontaneous glycinergic IPSCs in pharmacological isolation (with NBQX, D-APV, and bicuculline), as we did for alfaxalone concentrations at 10 μM or below. However, we did test the effects of 25 μM alfaxalone on spontaneous IPSCs (with NBQX and D-APV, but not bicuculline, Figure 1 and Table 1) and found significant decreases in spontaneous IPSC amplitude and frequency. We subsequently showed that spontaneous IPSCs in this latter condition were glycinergic, as amplitude and frequency were not significantly altered by the addition of bicuculline (Figure 2 and Table 2). Miniature IPSC frequency also decreased at alfaxalone concentrations at or above 3 μM, consistent with a decrease in activity-independent presynaptic glycine release probability. As alfaxalone reduced evoked IPSC amplitude from 30 nM upwards, the lack of effect on spontaneous IPSC amplitude at lower alfaxalone concentration may be due to the large variability in spontaneous IPSC amplitude, masking changes in amplitude. Other investigators have noted that both spontaneous and miniature glycinergic IPSCs have highly variable amplitude (Lim et al., 1999, 2003; Singer and Berger, 1999; Mitchell et al., 2007).

### Alfaxalone Causes Dose Dependent Reduction in Evoked IPSC Amplitude and Shape

Alfaxalone elicited a dose-dependent reduction in evoked IPSC peak amplitude at or above 30 nM, to 36% of control at 10 μM alfaxalone. This reduction in evoked IPSC amplitude could be due to either a presynaptic depression of glycine release, or postsynaptic reduction of GlyR activity. We also found that PPF of evoked IPSCs was significantly decreased at or above 3 μM alfaxalone. PPF is regulated by residual presynaptic calcium (Jackman and Regehr, 2017), and a decrease in PPF implies an increase in residual presynaptic calcium. Thus, in isolation, increases in presynaptic calcium should increase glycinergic neurotransmission, rather than a decrease in evoked glycinergic IPSCs, as we have observed. This makes interpretation of changes in PPF more complicated. By contrast, alfaxalone and other neurosteroids have been reported to decrease voltage-activated calcium currents, by interacting with binding sites on voltage-gated calcium channels (Ffrench-Mullen et al., 1994; Hirota and Lambert, 1996; Lambert et al., 1996; Nakashima et al., 1998; Kitayama et al., 2002), making an increase in presynaptic calcium unlikely.

Indeed, other effects of alfaxalone on evoked IPSCs are more consistent with modulation of postsynaptic GlyRs. Evoked IPSC rise-time was increased from 30 nM alfaxalone and evoked IPSC decay time was increased at 10 μM alfaxalone concentrations. Glycinergic IPSC rise and decay phases are limited by GlyR channel opening and closure, rather than by glycine rebinding or GlyR desensitization (Legendre, 1998; Singer et al., 1998; Singer and Berger, 1999). The rise-time of glycine-evoked currents resulting from recombinant mammalian α1 and α_1_β GlyR channel openings or native GlyRs in zebrafish neurons is glycine concentration-dependent, in that rise-time decreased with increasing glycine concentrations (Legendre, 1998; Mohammadi et al., 2003). A presynaptic reduction in glycine release therefore might result in delayed GlyR channel opening and therefore an increase in IPSC rise-time, consistent with a shift to a “reluctant” gating state which has been reported to contribute to the kinetics of miniature IPSCs mediated by GlyRs in zebrafish brain neurons (Legendre, 1998). However, the amplitude of miniature IPSCs is independent of their rise-time, consistent with a saturating concentration of glycine at postsynaptic GlyRs (Legendre, 1998). Thus, increases in evoked IPSC rise-time and decay time prolongation are consistent with postsynaptic modulation of the GlyRs, suggesting that alfaxalone increases the latency to opening of GlyRs by synaptically released glycine, and either delays GlyR closure or increases the probability of channel opening. One explanation for decreased evoked IPSC PPF is that alfaxalone either enhances glycine channel desensitization or deactivation, so that fewer glycine channels are available to respond to a second synaptic pulse of glycine. Clearly, single channel recordings from GlyRs would be needed to determine whether alfaxalone directly modulates GlyR activity.

Taken together, changes in the evoked IPSC amplitude and time course are suggestive of postsynaptic GlyR modulation by alfaxalone, leading to IPSC amplitude reduction and prolongation of IPSC rise and decay phase. Our results are most parsimoniously explained by direct modulation of GlyRs by alfaxalone. However, we cannot rule out the possibility that alfaxalone causes indirect modulation of GlyRs by activating other receptors, ion channels or signaling pathways.

### High Alfaxalone Concentrations Produce an Inward Current Without Altering Input Resistance and Potentially Activates Cation Channels

Our internal solution for recording both EPSCs and IPSCs was CsCl-based, with internal chloride reversal potential close to 0 mV (ie an inward current when holding at −60 mV). As high doses of strychnine block both GABA_A_ and glycine channels, the inward current generated by alfaxalone in the absence of strychnine (e.g., Figure 1) could be due to tonic activity of either channel. However, since other evidence presented here indicates that alfaxalone decreases glycine channel activity, we suggest that one source of inward current after alfaxalone application could be tonic GABA_A_ channel activity. The application of alfaxalone (1–100 μM) to bovine chromaffin cells elicits simultaneous activation of GABA_A_ channels and inhibition of nicotinic acetylcholine channels in whole cell patch clamp recording with a CsCl pipette solution, producing a net inward current through chloride-permeable channels (Cottrell et al., 1987). Although we used roughly equivalent recording conditions in these experiments, it is unlikely that all of this inward current was generated by activation of GABA_A_ channels, due to the presence of bicuculline in our external recording solutions when applying alfaxalone at concentrations less than 25 μM. We did observe an inward current in the presence of high strychnine concentration, when recording spontaneous EPSCs. It is notable that alfaxalone elicited an outward current in the presence of TTX, suggesting that modulation of the persistent sodium current might account for the inward current seen without TTX (van Zundert et al., 2008; Bellingham, 2013).

Alternate explanations for the inward current elicited by alfaxalone include modulation of the hyperpolarization-activated cation (I_H_) current. The I_H_ current is an inward rectifying cationic current that produces an increased inward membrane conductance during hyperpolarization from resting membrane potential, and, importantly, has been reported in rat HMNs (Bayliss et al., 1994; Berger et al., 1995). The effect of I_H_ activation is to depolarize the membrane potential until a potential is reached at which I_H_ is largely inactivated once more. Positive shifts in the voltage dependence of I_H_ can thus move resting membrane potential to more positive levels (Ireland et al., 2012; Wenker et al., 2012), while negative shifts in I_H_ voltage dependence or the absence of I_H_ can hyperpolarize resting membrane potential (Chen et al., 2005; Bellingham, 2013). The inward current caused by high alfaxalone concentrations could thus be due to a positive shift in the voltage activation of the I_H_ current; this mechanism would also be compatible with a lack of change in input resistance, because the slow activating I_H_ current is not directly assessed by the brief membrane pulses used to measure input resistance.

Alternatively, the lack of change in input resistance may be due to the inability of a somatic recording pipette to measure changes in input resistance originating from remote dendritic membrane. Evidence for this is seen in the largely dendritic excitatory synaptic inputs from the respiratory pattern generator, which produce a large change in conductance that is not associated with a change in input resistance (Rekling et al., 2000).

The overall results presented here suggest that clinically relevant levels of alfaxalone produce a reduction in glycinergic inhibition of MNs, by both presynaptic and postsynaptic mechanisms. Alfaxalone decreases glycine release from presynaptic terminals and also causes postsynaptic GlyR channel modulation, two mechanisms that, together, reduce the strength of glycinergic inhibition received by rat HMNs. As motor neuroexcitation is a common side effect of alfaxalone anesthesia in many species, pre-medications that can up-regulate glycinergic responses, examples of which include some α- and β-amino acids (Schmieden and Betz, 1995), propofol and ethanol (Chesnoy-Marchais, 1999), tamoxifen (Chesnoy-Marchais, 2005), and ivermectin (Shan et al., 2001), might be useful adjuncts in veterinary anesthesiology. In addition, etomidate, an intravenous anesthetic commonly used in humans, frequently causes excitatory movements and myoclonia (Doenicke et al., 1999; Harrison and Sear, 2004). It would be of great interest to see whether etomidate also produced a similar reduction in glycinergic neurotransmission to MNs.

How alfaxalone causes these changes in glycinergic neurotransmission remains to be determined. Recently pregnenolone, the precursor of all endogenous neurosteroids, has been shown to allosterically interact with cannabinoid receptors (Vallée et al., 2014), which, in turn, can modulate both presynaptic glycine release and GlyR function in HMNs (Lozovaya et al., 2005, 2011; Mukhtarov et al., 2005). It is possible that alfaxalone may also interact with cannabinoid receptors, which then reduce glycine release by the presynaptic terminal and/or postsynaptic GlyR activity.

Although our results provide the first direct evidence for neurosteroid-mediated reduction of glycinergic synaptic transmission to rat HMNs without affecting other factors regulating cell excitability, and are thus potentially applicable to other MNs which also receive strong glycinergic inputs, it remains to be seen if alfaxalone causes similar effects on glycinergic neurotransmission in other brainstem and spinal MNs. However, we believe our work sheds some light on the mechanism of neuromotor excitation observed with alfaxalone administration, and thus can help in designing better methods to block these effects.

## Data Availability

The datasets generated for this study are available on request to the corresponding author.

## Ethics Statement

This study was carried out in accordance with the recommendations of the Queensland Government Animal Research Act 2001, associated Animal Care and Protection Regulations (2002 and 2008), as well as the Australian Code for the Care and Use of Animals for Scientific Purposes, 8th Edition (National Health and Medical Research Council, 2013). The protocol was approved by The University of Queensland Anatomical Biosciences Animal Ethics Committee.

## Author Contributions

CL and PT conducted experiments. CL, PT and MB analyzed data, made figures, and wrote and edited the manuscript.

## Conflict of Interest Statement

The authors declare that the research was conducted in the absence of any commercial or financial relationships that could be construed as a potential conflict of interest.
