# Supplementary material for: Alfaxalone Causes Reduction of Glycinergic IPSCs, but Not Glutamatergic EPSCs, and Activates a Depolarizing Current in Rat Hypoglossal Motor Neurons
Source: Front Cell Neurosci. 2019 Mar 22;13:100. doi: 10.3389/fncel.2019.00100 (PMC6440435; doi:10.3389/fncel.2019.00100)
Supplement: Supplementary file 1 [file Table_1.DOCX]

**Supplementary information**

**Effects of alfaxalone (25 µM) on neuronal excitability**

| Alfaxalone  concentration | Action potential (AP) Amplitude (mV) | Maximum AP rise  slope (mV/ms) | AP Half-Width  (ms) | Maximum AP count |
| --- | --- | --- | --- | --- |
| Control | 111  (96.09-124.9) | 98.5  (88.4-108.6) | 1.46  (1.3-1.6) | 27.1  (21.5-32.8) |
| 25µM | 108  (89.2-127.3) | 97.5  (75.7-119.4) | 1.49  (1.3-1.7) | 25  (21-29) |
| *P* = | 0.68 | 0.89 | 0.59 | 0.12 |

Mean and 95% CI, n=8 HMNs. Paired two tailed t test.
